# Query Optimization for Distributed Spatio-Temporal Sensing Data Processing

**DOI:** 10.3390/s22051748

**Published:** 2022-02-23

**Authors:** Xin Li, Huayan Yu, Ligang Yuan, Xiaolin Qin

**Affiliations:** 1 College of Computer Science and Technology, Nanjing University of Aeronautics and Astronautics, Nanjing 211106, China; yuhycs@nuaa.edu.cn (H.Y.); qinxcs@nuaa.edu.cn (X.Q.); 2 State Key Laboratory for Novel Software Technology, Nanjing University, Nanjing 210023, China; 3 College of Civil Aviation, Nanjing University of Aeronautics and Astronautics, Nanjing 211106, China; yuanligang@nuaa.edu.cn

**Keywords:** spatio-temporal sensing data, spatio-temporal data processing, spatio-temporal index, polygon range query algorithm, *k* nearest neighbor query algorithm, query optimization, SpatialHadoop

## Abstract

The unprecedented development of Internet of Things (IoT) technology produces humongous amounts of spatio-temporal sensing data with various geometry types. However, processing such datasets is often challenging due to high-dimensional sensor data geometry characteristics, complex anomalistic spatial regions, unique query patterns, and so on. Timely and efficient spatio-temporal querying significantly improves the accuracy and intelligence of processing sensing data. Most existing query algorithms show their lack of supporting spatio-temporal queries and irregular spatial areas. In this paper, we propose two spatio-temporal query optimization algorithms based on SpatialHadoop to improve the efficiency of query spatio-temporal sensing data: (1) spatio-temporal polygon range query (STPRQ), which aims to find all records from a polygonal location in a time interval; (2) spatio-temporal *k* nearest neighbors query (ST*k*NNQ), which directly searches the query point’s *k* closest neighbors. To optimize the ST*k*NNQ algorithm, we further propose an adaptive iterative range optimization algorithm (AIRO), which can optimize the iterative range of the algorithm according to the query time range and avoid querying irrelevant data partitions. Finally, extensive experiments based on trajectory datasets demonstrate that our proposed query algorithms can significantly improve query performance over baseline algorithms and shorten response time by 81% and 35.6%, respectively.

## 1. Introduction

With the development of Internet of Things (IoT) technology and the proliferation of mobile smart devices, a large amount of spatio-temporal sensing data have been generated continuously by diverse applications, such as GNSS-enabled mobile devices [1,2], urban traffic [3,4], satellites, and various sensing devices [5,6]. Continuous, consistent, and long time-series data from remote sensing are essential to monitor changes in geolocation information. The derived datasets have the advantage of extensive spatial and temporal coverage. Accurately extracting these sensing data can significantly give impetus to data mining [7,8] and data prediction [9]. For example, storing the speed and direction of moving objects in a spatio-temporal database can predict the location, which can assist spatio-temporal applications such as urban management [10,11], traffic control [12], and route planning [13]. Location-based social networks (LBSNs) can extract and analyze large amounts of user location information to provide geomarketing and recommendation services [14]. This paper is an extended version of our conference paper: Huayan Yu, Xin Li, Ligang Yuan, Xiaolin Qin. “Efficient Spatio-Temporal-Data-Oriented Range Query Processing for Air Traffic Flow Statistics.” Proceedings of 2021 IEEE Intl Conf on Parallel & Distributed Processing with Applications, Big Data & Cloud Computing, Sustainable Computing & Communications, Social Computing & Networking (ISPA/BDCloud/SocialCom/SustainCom), New York, USA, 30 September–3 October 2021.

However, processing such datasets is often challenging due to complex sensor data geometry characteristics, anomalistic spatial region, and so on. The spatio-temporal processing operation refers to the analysis of data with temporal attributes and relative positions in three-dimensional space based on the spatio-temporal data models [15]. Enormous spatio-temporal data objects are unique in terms of time and space attributes. They are characterized by high dimensionality, spatio-temporal dynamic correlation, and multiple semantic operations, which intensely burden data processing work in efficient responding data queries. Existing spatial processing systems do not support spatio-temporal data analysis since they lack support for spatio-temporal data types and operators. The spatio-temporal processing framework must perform preprocessing operations such as data cleaning, segmentation, and compression on the original dataset. It also concludes the execution of spatio-temporal query and similarity matching operations based on the construction of spatio-temporal indexing.

Timely and efficient spatio-temporal query plays a significant role in improving the accuracy of data processing, providing assistance to support the efficient extraction of valuable information. Among those data processing operations, the spatio-temporal query is one of the most basic but time-consuming operations. Firstly, the query time cost is high. A spatio-temporal query usually involves a series of distance-based queries and data aggregation operations, demanding a lot of storage and computing resources. It often requires access to the entire spatio-temporal database for retrieval query results [16]. Secondly, the query pattern is unique due to the complex structure and high dimension of data, for example, to query the flow of people or traffic in a particular area for a period of time or to query the recommended service information of nearby stores near a query point [17]. These queries are usually not efficiently supported by traditional inverted or B+ tree indexes. B+ tree is a variant of B tree, whose non-leaf nodes only contain index information. B+ tree index has better spatial locality, so that it can efficiently support queries such as single-dimensional numerical ranges, but it is difficult to support high-dimensional queries on spatio-temporal data. In order to efficiently match query requests in different scenarios, it is necessary to provide more creative query methods.

However, existing query algorithms show their weakness in three parts: (1) Existing research lacks support for multidimensional spatio-temporal queries. The current query and processing algorithms [18,19,20] only consider the spatial dimension, ignoring the temporal information, and are only suitable for geospatial scenarios. However, users are more concerned about the location relationship that changes dynamically over time; (2) The expansion ability under the complex spatial region model is insufficient. The research [21,22,23] only supports the rectangular spatial model, which rarely pays attention to the problem of range query under complex irregular polygonal shapes (such as city boundaries and delivery areas). These regions usually have characteristics such as large boundaries and irregular shapes and often require a large number of vertices to be accurately represented in a vector-based form; (3) The efficiency of the spatio-temporal query needs to be further improved and optimized. Due to the ubiquitous spatial correlation in spatio-temporal data, coupled with the randomness and complexity of the time dimension, the spatio-temporal query algorithm needs to have high execution efficiency. Existing query optimization technologies [24,25] are mainly based on spatio-temporal index to improve query performance. Plentiful indexing techniques for spatio-temporal data have been proposed to improve the performance of spatio-temporal queries (such as R-tree [26], quad-tree [27], and KD-tree [28]). However, they are not optimized to deal with large-volume spatio-temporal data covering high-dimensional features with high performance.

Motivated by these observations, in order to consider the multidimensional spatio-temporal query, support the expansion of complex spatial region models, and further improve the query response performance of massive data, we propose two spatio-temporal range query MapReduce algorithms based on SpatialHadoop to analyze spatio-temporal data efficiently and effectively. We extend SpatialHadoop by designing a parallel spatio-temporal polygon range query (STPRQ) algorithm and the spatio-temporal *k* nearest neighbor Query (ST*k*NNQ) algorithm. The contributions of this paper are summarized as follows:

- We propose a distributed spatio-temporal polygon range query algorithm STPRQ. The algorithm proposes a polygon range query model based on the global index in the spatial range search stage and refilters the data objects under the spatial and temporal constraints based on the record reader.
- We propose a spatio-temporal k nearest neighbor algorithm STkNNQ, which comprehensively considers the temporal and spatial factors to calculate the spatio-temporal proximity. To improve query efficiency, we propose a spatio-temporal data partition strategy based on the global index. We also propose an adaptive iterative range optimization (AIRO) strategy, which can optimize the iterative range of the algorithm to avoid the time cost caused by querying irrelevant data blocks.
- We conduct extensive experiments on real-world aviation trajectory datasets to evaluate the efficiency and effectiveness of our proposed query algorithms. The experimental results show that the STPRQ algorithm can improve query efficiency by reducing the query cost to 19%. The experimental results also indicate that the STkNNQ algorithm can improve the query efficiency of spatio-temporal data, shortening the response time by 35.6%.

## 2. Related Work

### 2.1. Spatio-Temporal Data Management and Processing

Early research mostly focuses on spatio-temporal data models [29], spatio-temporal query languages [30], and query processing and optimization. In recent years, some generic database systems (such as SECONDO [31], Oracle Spatial, and PostGIS) have been designed so that we can store, query, and manage spatio-temporal data. To optimize query efficiency, scholars have turned their attention to designing indexing techniques to index spatio-temporal data [26,27,28]. Theodoridis et al. [32] proposed an indexing scheme 3D-R tree, which regards the time dimension as the third attribute dimension besides latitude and longitude. However, it is not suitable for storing large timespans of data. Tao et al. [33] proposed an extension to the 3D-R tree to make filtering better. Most of these explorations are based on spatio-temporal data systems run on single-node servers and encounter scalability bottlenecks when in tremendous data scenarios.

Some attempts have been made to improve the efficiency of spatio-temporal data management in the distributed environment. The existing solutions for spatial data can be classified as Hadoop-based systems and Spark-based systems, which are mostly built on top of distributed spatial data management systems (DSDMSs). The extremely outstanding contributions in the context of Hadoop-based DSDMSs are the following research prototypes: SpatialHadoop [18], Hadoop-GIS [19], and HadoopTrajectory [34]. However, as mentioned above, all techniques mostly focus on processing traditional spatial data such as rivers, railways, and buildings. ST-Hadoop [35] is an extension of SpatialHadoop [36] that integrates spatio-temporal concepts in each layer of SpatialHadoop. Thus, it can support spatio-temporal range queries and joins. Although these systems exhibit high scalability data processing operation, Hadoop will involve heavy IO access from the disk.

Due to the high IO cost in Hadoop, some systems based on Spark are proposed [20,21,22,37]. GeoSpark [20] extends Spark for processing spatial data. Simba [21] offers scalable and efficient in-memory spatial queries for big spatial data. The STARK [22] framework adds spatio-temporal support to Spark, including spatial partitioners and several models for indexing. All spatial data systems above share a drawback: they are all not suitable for performing spatio-temporal operations. A possible explanation is that their indexes are only for processing spatial operations and cannot identify the characteristics of spatio-temporal data.

### 2.2. Distributed Spatio-Temporal Query

The spatio-temporal query is a typical distributed spatio-temporal processing and analysis method. The spatio-temporal query operations include range query, *k* nearest neighbor (kNN), *k* closest pair query, and distance join query. Table 1 depicts the most representative systems with functional comparisons. As we can see, range query and kNN query are the most common operations, while most distributed data management systems do not fully support the expansion in spatio-temporal dimensions. They show a lack in supporting multi-dimensional spatio-temporal queries and complex spatial regions. This paper mainly focuses on the query optimization research of range query and kNN query.

#### 2.2.1. Spatio-Temporal Range Query

Spatio-temporal range query is generally used in spatial databases to query data in complex geometric regions to determine a set of candidate spatial objects that may satisfy the query conditions. Related research works [10,18,35] introduce spatial indexes and implement range query algorithms for processing spatio-temporal objects. Chen et al. [24] designed a built-in index structure STEHIX for HBase to handle spatio-temporal queries. Oh et al. [25] extended the standard range query algorithm to moving objects and made improvements and optimizations, and proposed an effective method for range space keyword query. Nevertheless, these methods only support the rectangular spatial model, which rarely pays attention to the range query problem under complex irregular polygonal shapes (such as city boundaries and delivery areas).

There are few studies for spatio-temporal polygon range query processing, where historical trajectories are used to predict the possibility that a moving object will move towards the following polygon location. The challenge is that accurate polygon queries require high data correlation and a compact representation of query range, whereas spatial indexes to filter data are coarse-grained. Zacharatou et al. [38] converted a polygon aggregation query into a set of drawing operations on a canvas and provided accurate results when combined with a polygon index. Zhang et al. [39] designed and developed an end-to-end system on graphics processing units (GPUs) to associate points with the polygons by utilizing the massive data-parallel computing power of GPUs. In the paper [12], we integrated the spatio-temporal polygon range query algorithm to solve the problem of traffic flow statistics for civil aviation airspace. We will further optimize the spatio-temporal polygon range query method proposed. In conclusion, the expansion ability under the complex spatial region model is insufficient, so it is urgent to make our design for a spatio-temporal polygon range query.

#### 2.2.2. Spatio-Temporal *k* Nearest Neighbor Query

**Figure 1:**
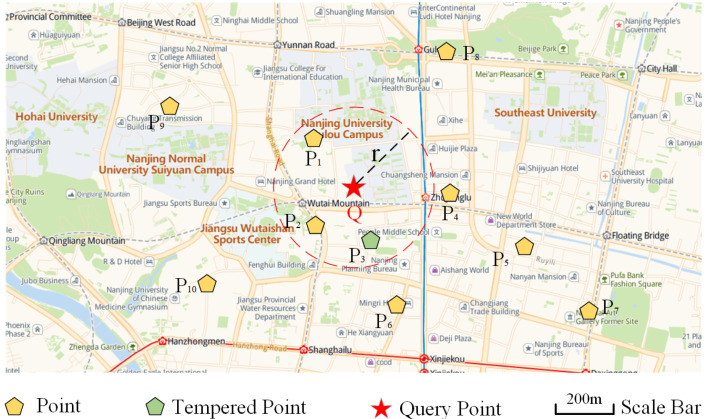
The scenario of spatio-temporal k nearest neighbors query [44]. Given a set of check-ins of spatio-temporal points, kNN (*k* = 3) finds the nearest points of query point *Q*. The *r* denotes the minimum extension radius of query range that contains exactly *k* results. If we consider spatial closeness only, we will obtain three points for *Q*, i.e., $P_{1}$, $P_{2}$, and $P_{3}$. However, if we consider temporal concurrency as well, $P_{3}$ may no longer be the *k* nearest to *Q* when it is outdated.

As can be seen from Figure 1, the spatio-temporal *k* nearest neighbor query calculates the temporal proximity and spatial proximity and returns *k* data objects closest to the query point. The *k* nearest neighbor query algorithm has been extensively studied in the literature. However, most existing solutions for *k*NN consider the spatial closeness only. They either ignore temporal concurrency or suffer from poor query efficiency. Chen et al. [24] designed a two-layer index structure based on HBase and proposed a load balancing and scalable kNN query. Some research works also consider the problem of *k*-nearest neighbor join query between two datasets. García-García et al. [40] elaborated and compared the existing distance join query work. Using the repartitioning technology of dense spatial regions, they improved the *k* nearest neighbor join query algorithm based on MapReduce and extended the spatial object to more complex objects such as polygons or line segments. Zhang et al. [41] designed a MapReduce-based k-nearest neighbor join query, used pruning rules to filter spatial distances, and proposed two approximate algorithms to minimize the number of replicas. Liu et al. [42] extended the kNN method based on the MapReduce framework but only considered the spatial proximity and ignored the temporal proximity. For considering both spatial and temporal proximity, Li et al. [43] proposed the ST-kNN algorithm. In order to achieve an effective ST-kNN connection, this study also proposes a time range count index (TRCindex) to reduce the data transmission overhead between different machines. The above analysis shows that the current research work has not solved the spatio-temporal proximity in the *k* nearest neighbor query problem, and the query efficiency needs to be further optimized. Our previous research [12] integrates the spatio-temporal *k* nearest neighbor algorithm to SpatialHadoop, which supports spatio-temporal proximity but is still not efficient enough. We will further optimize query models in this paper.

## 3. Problem Preliminaries

In this section, we first define spatio-temporal data. After that, semantic details of respective range query algorithms and the corresponding notation and processing paradigms are presented below.

### 3.1. Spatio-Temporal Data

In order to formally describe the entire spatio-temporal range query, the definition of spatio-temporal data is given first. Spatio-temporal data usually refers to a collection of location records marked with timestamps, which can be any data type (such as a point, a line string, etc.). Spatio-temporal data can provide accurate and comprehensive general information, containing two parts: (1) spatio-temporal information, including a spatial attribute and a time attribute; and (2) other properties, including height, speed, angle information, etc. Table 2 is an example of spatio-temporal record. Using the millions of spatio-temporal records continuously uploading from the Hadoop Distributed File System (HDFS), we can carry on different range queries in a spatial area within a temporal range.

**Definition** **1** (ST-point)**.** *A spatio-temporal dataset is defined as a collection $P=\{p_{1},p_{2},\ldots ,p_{n}\}$. For each ST-point (spatio-temporal point), p=(lng,lat,t), lng represents longitude, lat represents latitude, t represents timestamp, and δ means other attributes information.*

### 3.2. Spatio-Temporal Polygon Range Query

**Figure 2:**
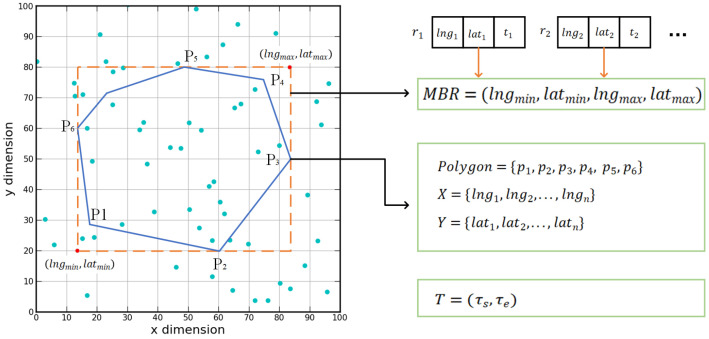
Description of the query condition of spatio-temporal polygon range.

To describe the spatial region range, a characterization of the polygonal region model is given. As shown in Figure 2, the bounding area of a polygon is defined by the coordinates of a series of spatial vertices and the line segments connecting the coordinate points. Let $P=\{p_{1},p_{2},\ldots ,p_{n}\}$ be a set of spatio-temporal points in $E^{d}$ (*d*-dimensional Euclidean space); for each p∈P, we have p=(lng,lat,t). A query range of polygon Q=(S,T) contains a spatial range *S* and a time range *T*, where *S* is a shape of polygon, and $T=(\tau_{s},\tau_{e})$ is a time range, and $\tau_{s}$, $\tau_{e}$ denote the start and end time of the interval. The timespan of *p* is defined as $\left|T\right|=\tau_{s}-\tau_{e}$. Polygon query range *S* consists of a set of spatial points forming its boundary, which is represented by a positive number *n*, a set of *x*-axis coordinates $X=\{lng_{1},lng_{2},\cdots ,lng_{n}\}$, and a set of *y*-axis coordinates $Y=\{lat_{1},lat_{2},\cdots ,lat_{n}\}$, where lng, lat denote the longitude and latitude coordinates of a point. Each pair of (lng,lat) defines a vertex of the polygon. Then, the first and final pairs of (lng,lat) points are joined by a line segment that closes the polygon.

**Definition** **2** (MBR)**.** *The MBR (minimum bounding rectangle) is the smallest axis-aligned rectangle containing all query range points. We define the MBR of the polygon as the maximum range of the polygon expressed in two-dimensional coordinates, which can be represented by two points MBR = <($lng_{min},lat_{min}),(lng_{max},lat_{max}$)>. Compared with directly searching the spatial relationship of spatial objects, finding MBR is simpler and more efficient.*

**Definition** **3** (STPRQ)**.** *Given a spatio-temporal dataset, a polygonal range, and a temporal range, the STPRQ finds all the points of the dataset that fall within the polygonal shape, comprising a list of line segments. The spatio-temporal polygon range query is formulated as*

(1) STPRQ(P,S,T)={p∈P|p∗MBR∧p⋄S∧p.t∈T}

(2) $$p*MBR=\{p\in P|\exists p\in P,lng_{min}\leq p.lng\leq lng_{max}\wedge lat_{min}\leq p.lat\leq lat_{max}\}$$

(3) p⋄S={p∈P|∃p∈P,S.PNPOLY(p.lng,p.lat)}

(4) $$p.t\in T=\{p\in P|\exists p\in P,\tau_{s}\leq p.t\leq \tau_{e}\}$$

*Here, PNPOLY() (point inclusion in polygon test) means the classic algorithm to find if a point lies within a polygon.*

### 3.3. Spatio-Temporal *k* Nearest Neighbor Query

The ST*k*NNQ is one of the most important and studied spatio-temporal operations. For example, when an airplane encounters an emergency, it obtains a communication connection by immediately calculating the *k* nearest neighbors with other airplanes. The spatio-temporal *k* nearest neighbor query discovers the *k* closest points to a given query point (i.e., it reports only the top *k* points). To locate spatio-temporal points and calculate the distance between points, we use Euclidean space to calculate the distance for simplicity. The formal definition of the ST*k*NNQ for points is as follows:

**Definition** **4** (ST*k*NNQ)**.** *Let $P=\{p_{1},p_{2},\ldots ,p_{n}\}$ be a set of spatio-temporal points in $E^{d}$ (d-dimensional Euclidean space), a query point q in $E^{d}$, a positive number $k\in N^{+}$, a spatio-temporal predicate $\theta (\theta_{space},\theta_{time})$, and a spatio-temporal sorting function $F_{\alpha }$; the STkNNQ returns a set of spatio-temporal data $P^{{}^{\prime }}\subseteq P$, and $|P^{{}^{\prime }}|=k$, i.e., the k closest points to q. For each point $p_{i}\in P^{{}^{\prime }}$, $F_{\alpha }\left(q,p_{i}\right)\leq F_{\alpha }\left(q,p_{j}\right)$, that is,*

(5) $$STkNNQ\left(P,q,\theta ,F_{\alpha },k\right)=\{p_{i}\in P^{{}^{\prime }}|P^{{}^{\prime }}\subseteq P\wedge \forall p_{j}\in P\backslash P^{{}^{\prime }},F_{\alpha }\left(Q,p_{i}\right)\leq F_{\alpha }\left(Q,p_{j}\right)\}$$

*where $p_{i}$ denotes the i-th point which belongs to dataset $P^{{}^{\prime }}$, and $p_{j}$ denotes the j-th point which belongs to dataset P but does not belong to $P^{{}^{\prime }}$. The k points also satisfy that they are included by $\theta_{time}$ and $\theta_{space}$. For the spatio-temporal sorting function $F_{\alpha }$, the definition is as follows:*

(6) $$F_{\alpha }\left(q,p\right)=\left\{\begin{array}{cc} f_{s}\left(q.loc,p.loc\right) & \alpha =1\\ \alpha \times f_{s}\left(q.loc,p.loc\right)+\left(1-\alpha \right)\times f_{tl}\left(q.time,p.time\right) & 0\leq \alpha \leq 1\\ f_{t}\left(q.time,p.time\right) & \alpha =0 \end{array}\right.$$

The spatio-temporal sorting function $F_{\alpha }$ indicates whether the query user-defined is more inclined to spatial proximity or temporal proximity. The $F_{\alpha }$ combines the spatial proximity and spatio-temporal proximity of each point to the query point and sorts in turn. α=1 denotes that users focus more on spatial proximity, and α=0 denotes that users are more concerned about temporal proximity. In order to locate the distance between objects and query objects in space, this paper uses Euclidean distance to measure the distance between objects, which can fully reflect the mutual distance between objects. The distance between two points in space is expressed as

(7) $$f_{s}\left(q.loc,p.loc\right)=\frac{dist(q,p)}{\theta_{space}}\;$$

Here, dist(q,p) denotes the Euclidean distance between *p* and *q*.

(8) $$f_{t}\left(q.time,p.time\right)=\frac{\Delta (q,p)}{\theta_{time}}\;$$

Here, Δ(q,p) denotes the time deviation between *p* and *q*.

## 4. Query Processing Algorithms

### 4.1. Spatio-Temporal Polygon Range Query

#### 4.1.1. The Framework of Spatio-Temporal Polygon Range Query

The framework of our proposed solution for STPRQ contains two main steps: (1) spatial range search and (2) refilter and refine.

**Figure 3:**
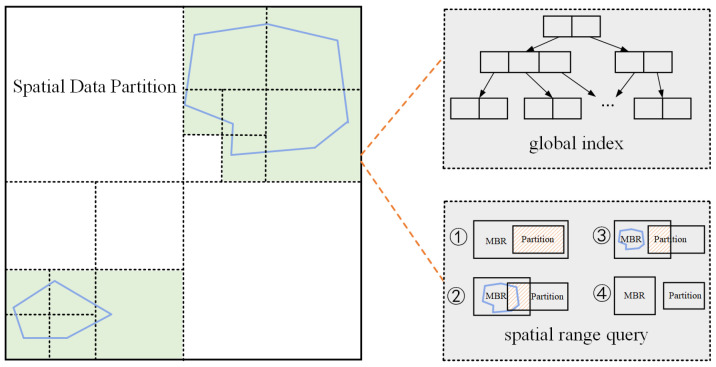
The process of polygonal spatial search. Each spatial partition is a data node of the global index. A polygon query range may spatially overlap with multiple data partitions.

- Spatial range search. Spatial search mainly performs a spatial range query on each matching partition and filters data that is not within the spatial range to select data blocks that intersect the query range. We use the global spatial index based on SpatialHadoop to partition the data block, such as grid index, R-tree index, quad-tree index, KD-tree index, space-filling curve, etc. The purpose of a global index is to store spatially adjacent data together to satisfy the principle of spatial locality. Although the space division ideas of diverse indexes are different, their essence is to use different space division algorithms to maximize the preservation of space characteristics and provide fast and efficient query efficiency.
  As shown in Figure 3, we regard the spatio-temporal dataset as points distributed in the spatial area with time attributes, then build an index for spatial data partitioning. Each index node can be regarded as a uniform data partition, of which the border is a rectangle. Since the boundary information of the partition of the data block is stored on the node of the global index, it is easy to judge the intersection of the partition and the polygon or the MBR of the polygon by using the global index.
  In combination with the query range for the index metadata, we clip data blocks to filter out all blocks that do not contain the records required by the query information. Since the location coordinates of data change dynamically with time, the data distribution still exhibits uneven characteristics. Therefore, using the spatial pruning strategy, coarse-grained filtering results can be obtained and passed to the next stage for execution.
- Refilter and refine. The spatial range search phase cannot guarantee that every record in the data block is within the query space and time query period. Therefore, it is necessary to perform refiltering and refining for each data block collected after pruning.
  In each spatial search step, we use the built-in SpatialRecordReader of SpatialHadoop to traverse the data blocks obtained and compare each record’s time attribute with the query’s time interval to select records that match exactly. This step is essential because when a partition is selected, some areas may overlap with the query interval instead of being wholly included in the time interval, so the records need to be refined to delete for records that are not within the time interval. Once the data blocks within the spatial query range are selected, we will filter each matched data block for the precise temporal and spatial range. Finally, we verify whether the queried spatio-temporal records meet the conditions given by the user.

#### 4.1.2. Spatio-Temporal Polygon Range Query Algorithm

Algorithm 1 provides the pseudocode for polygonal range query operation. The input includes a spatio-temporal dataset *D*, where each object o∈D is represented by (lng,lat,t), a time interval $T=(\tau_{s},\tau_{e})$, a polygon query range *S*, and a range query result Res. Each $r_{i}$∈Res is satisfied such that the location of $r_{i}$ lies inside polygon range *S*.   
 **Algorithm 1:** STPRQ MapReduce Algorithm 

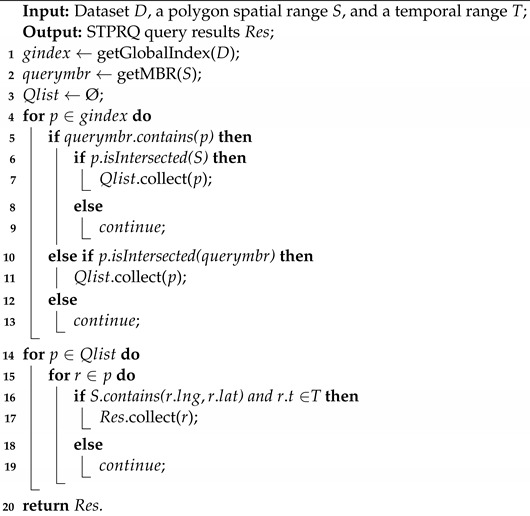



First of all, we initialize a set of parameters of the polygon range query. We use the global index to limit file size. Then, we obtain the minimum bounding rectangle (MBR) of the polygon query range. After that, an ordered collection is created to store the results of partitions that need to be processed by a MapReduce job. Next, we process partitions located in the query range. There are two cases: (1) the partition that is wholly contained in the MBR and intersects the polygon query range *S*; (2) the partition that both intersects with the MBR and intersects with *S*.

In general, partitions that are entirely contained in the query range should be processed and copied to output directly. In this way, we can filter out data partitions that do not contain query results. Finally, we check and filter all the records in the partitions and return records in the polygon area *S* and time range *T*.

Compared with the expensive logarithmic or even the power time complexity offered by traditional solutions, our range query achieves $O(\frac{n\varepsilon }{m})$ expected time, where *n* is the number of conditional partitions overlapping with the polygon query range, ε is the number of records in a partition, and *m* denotes the number of map tasks completed by each node. Regardless of the spatial indexes, the number of partitions, *N*, is defined by

(9) $$N=\lceil \frac{S(1+\alpha )}{B}\rceil$$

where *S* is the input files size, *B* is the HDFS block capacity, and α is an overhead ratio, set to 0.2 by default, which accounts for the overhead of replicating records and storing local indexes.

### 4.2. Spatio-Temporal *k* Nearest Neighbors Query

#### 4.2.1. The Framework of Spatio-Temporal *k* Nearest Neighbors Query

In distributed environments for ST*k*NNQ, it is vital to design a good data partition strategy, which requires the following: (1) Spatio-temporal proximity. Objects that are close spatially and temporally should be assigned to the same partition as much as possible. (2) Even distribution. The numbers of objects in different partitions are as equal as possible; thus, we can achieve load balance.

**Figure 4:**
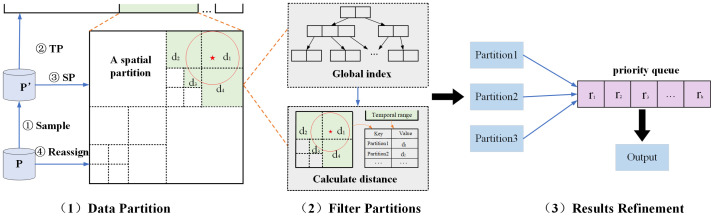
Overview of ST*k*NNQ algorithm.

Figure 4 presents the framework of our proposed solution for ST*k*NNQ, which consists of three main steps: (1) data partition, (2) filter partitions, and (3) results refinement.

- Data partition. This paper devises a simple but effective spatio-temporal data partition strategy. The partitioning stage is divided into four steps: sampling, time partition, spatial partition, and reassignment. During the sampling phase, a set of random samples are drawn from the dataset at a sampling rate of η=1%. Since it is randomly sampled from the original dataset, it maintains its spatio-temporal distribution characteristics. In the spatial partition step, we divide the global spatial range into multiple disjoint data partitions with clear boundary information. The quad tree is generally used to divide the global spatial domain in this paper. Compared with other indexes, quad tree considers all parts of the spatial domain, which can alleviate the problem of unbalanced spatial distribution and make it easier to divide the space. The minimum enclosing rectangle MBR of each object is adopted in this paper because checking the spatial relationship of two MBRs is much faster than checking the spatial relationship of the two records. In the reassignment phase, the global index generated based on the data samples is broadcast to each partition, and all datasets are traversed. For each p∈P, if *p* intersects with some partition of the global index, we inspect the record and update the boundary identifier of the current partition. At last, we repartition according to the bounded identifier.
- Filter partitions. In this step, with the global index, we can query and filter partitions based on the latitude and longitude of the point to be queried. First, we construct the MBR of the query point *q*, then calculate the distance from each partition to *q* according to the global index, and then obtain the time range $\theta_{time}$, spatial range $\theta_{space}$, and sorting function $F_{\alpha }$ according to the input, and the priority of the partition can be obtained. When the number of existing results is less than *k*, the partition will be selected from the remaining partitions for processing according to the sorting priority.
- Results refinement. After filtering the query partitions, each data partition containing objects that meet the conditions is obtained. This step is mainly to solve the problem of fully considering the time factor in the conventional spatial *k* nearest neighbor query and improving the query algorithm’s efficiency. Firstly, we scan the partitions to be processed, filter them by time range and spatial range, and deduplicate the duplicate records. Then, the results are redivided into new partitions. Finally, the local results of each partition are merged into global results.
  A priority queue is then constructed to prioritize each record according to a user-supplied spatio-temporal sorting function. If the results do not satisfy *k*, it will go back to the second step to continue the diffusion search; if *k* is satisfied, it is necessary to judge whether the nearest *k* points are already in the result set. Due to the density of data and the influence of the global index, other untraversed partitions have likely qualified records. Therefore, defining a query test area is necessary to reconfirm whether the *k* records in the result set are the final result. If the delineated test circle area intersects with other data partitions and the data block has not been processed before, a range query needs to be restarted to scan the data block to obtain closer results.

#### 4.2.2. ST*k*NNQ MapReduce Algorithm

Algorithm 2 gives the pseudocode of the *k*NN query algorithm. The basic idea behind the query processing algorithm for ST*k*NNQ is a four-step approach:

Generate the initial answers (lines 6–13). This step calculates the initial answers of k closest points to *q*. We first use a spatial range query to find all the partitions overlapping with *q*. Note that we may fail to find a matched partition. In this case, we trigger a kNN query to find the nearest partition. Then, we add all partitions to the priority queue. After that, a conditional matching on the partitions is performed within the priority. When $dist_{p}$ is less than the current $dist_{k}$, the partition will be ejected from the priority queue. Finally, each record in the partition whose time in the temporal range *T* will be traversed. This process will produce *k* initial results.

Check the correctness (lines 14–17). This component will check whether the *k* initial results are the final results. We calculate the new test range *C*, which is a circle centered at the query point *q*, and distance to the $k_{th}$ neighbor as radius. If *C* does not intersect with other partitions, then the *k* initial results are final, and cq is directly returned. Otherwise, the final results must be determined.

Determine the final results (lines 18–21). A spatial range query is triggered to obtain all the points insides the test circle *C* and back to the loop for several iterations. Finally, we determine the *k* points closest to the query point.

Initialization (lines 1–4). This step initializes some variables. The rq priority queue is used to store candidate records that meet the kNN condition. The pq is a priority queue that records all data blocks to be traversed. We use $dist_{k}$ to store the kth shortest distance from the query point *q* in the rq queue. We define the distance from the query point *q* to the partition *p* as the minimum distance from the point to the partition, which is defined as

(10) $$dist_{p}\left(p,q\right)=\min_{i\in p}dist\left(i,q\right)$$

The time complexity of the ST*k*NNQ algorithm is $O(\frac{nmt}{\omega }logk)$, where *n* is the number of selected partitions overlapping with the query point, *m* is the number of points in a partition, *t* is the number of iterations, *k* means the maximum heap of size, and ω is a factor that depends on different indexes. The number of iterations represents that the job may need multiple iterations when the total number of results is less than *k* in one iteration. Note that the optimal time complexity of the ST*k*NNQ algorithm is $O(mlog^{k})$, where the number of iterations is 1. Using our proposed approach, a significant reduction of the consumed computation time is obtained because the number of partitions decreases, which means fewer mappers.
 **Algorithm 2:** ST*k*NNQ MapReduce algorithm 
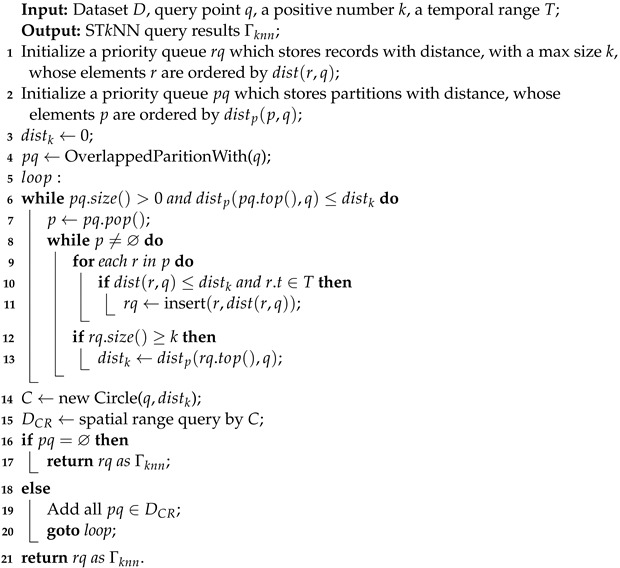


#### 4.2.3. Adaptive Iterative Optimization Algorithm

In order to reduce query time, it is crucial to design the scope of iterative searches elastically. Due to the different sparsity of data blocks, when the query point is in a sparse area, diffusing the search with a fixed iterative range will result in fewer data satisfying the query in the same spatial range. Therefore, the search cost of a new round of iteration is increased.

One of the most expensive overheads in a distributed environment is the data transmission among different machines, which is triggered when we query data blocks. Due to the limitation of the MapReduce framework, it usually takes much time for the map task to scan each data partition. The ST*k*NNQ algorithm expands the radius of the iteration range in each iteration, resulting in unnecessary data blocks being queried in the map phase. Therefore, the new iteration range will repeatedly scan the already queried data blocks, resulting in a sizable total response time.

In this paper, we propose an adaptive iterative range optimization algorithm (AIRO) considering data distribution, which can jointly consider the characteristics of data distribution and spatio-temporal query range and improve the overall stability for the ST*k*NNQ algorithm. We take the time range, spatial range span of the dataset, and query range into account, then generate a distance function to set the iteration. We make a trade-off between the number of iterations and query data blocks based on the features.

**Figure 5:**
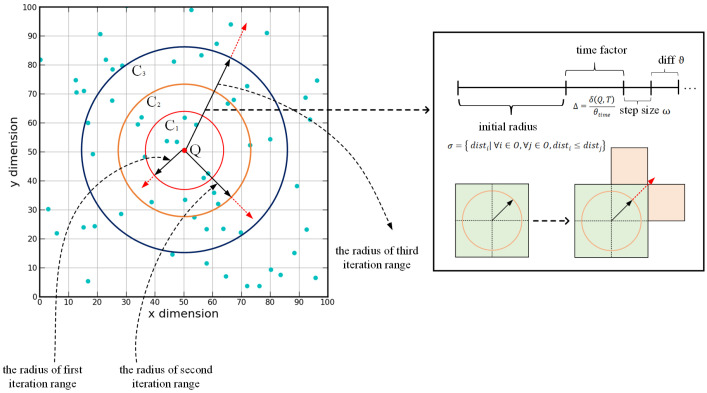
Overview of AIRQ algorithm. The iteration range for each round is a test circle of increasing radius, with each iteration overlapping new data records.

The goal of this algorithm is to converge the test circle radius as soon as possible, reduce the overhead of querying data blocks, and ultimately improve the algorithm’s performance. As shown in Figure 5, at first, the algorithm generates an initial iterative range based on the results of the first iteration range. Each time a new round of diffusion search is performed, the iteration range radius is expanded based on the original iteration range. Assuming that three expansions are required to obtain the final result set, the iterative range radius of three rounds needs to be generated, respectively. The primary factors are as follows:

1. Set the initial iteration range radius and step size. We select the farthest object from the query point *q* in the intermediate results *O* generated by the previous iteration range and calculate its distance σ to the *q*
  (11) $$\sigma =\{dist_{i}|\forall i\in O,\forall j\in O,dist_{i}\leq dist_{j}\}$$
  and set the initial iteration range radius gr=σ∗β, and the initial step size gs=σ∗0.001. The reason for this setting is that all objects in the previous iteration range may not satisfy the query conditions so that the obtained result set is farther away from the query point than the actual distance.
2. Calculate the time impact factor. The larger Δ is, the smaller the influence on the iteration range.
  (12) $$\Delta =\frac{\delta (q.t,T)}{\theta_{time}}\;$$
  δ(q.t,T) denotes the difference between the query point *q* and the time range *T*.
3. Generate the new radius of the new iteration range. We take the initial iteration range gr, and combining the time influence factor Δ, and taking the step size gs into account, the radius of the new iteration range is finally generated.

## 5. Experiment Results and Analysis

### 5.1. Experimental Datasets and Setup

#### 5.1.1. Datasets

To evaluate the experimental performance of our algorithms, we use a real-world spatio-temporal dataset and a synthetic dataset. (1) ADS-B trajectory dataset. The dataset contains the 10 million records of automatic dependent surveillance-broadcast (ADS-B) trajectory data collected by radar services. The ADS-B trajectory messages can provide more accurate and comprehensive general information, which contain two parts of information: (1) spatio-temporal information, which includes longitude, latitude, and a timestamp; and (2) other properties, including flightId, height, sector information, etc. (2) Synthetic dataset. The dataset is generated by sampling and copying the trajectory dataset up to 100 million records to test the performance of performing parallel computing. More details about these datasets can be found in Table 3. Furthermore, the parameters used in the queries are described in Table 4.

#### 5.1.2. Experimental Settings

Server specification. To ensure the high availability of experiments and nonvolatility of data copies stored on HDFS, all our experiments are carried out on a cluster of six nodes, including one master node and five slave nodes. Each node is configured as follows: CentOS-7, 8-core CPU, 16 GB RAM, and 80 GB disk. The block size of each node is 64 MB, and the replication factor is three.

Testing environment. We choose ST-Hadoop as the comparison system because ST-Hadoop is also based on SpatialHadoop, which supports both spatio-temporal range query and spatio-temporal kNN query. To verify the effectiveness of the indexing strategy, we select four indexes for horizontal comparison, namely *str*, *grid*, *quad*, and *kdtree*. Correspondingly, we choose three different time-slicing strategies of ST-Hadoop for comparison, i.e., year, month, and week, based on spatial index named *str*.

### 5.2. Experimental Analysis of Spatio-Temporal Polygon Range Query

#### 5.2.1. Performance of Spatio-Temporal Polygon Range Query

The spatio-temporal range parameter as specified in Table 4 is set. The dataset size is set from 20% to 100% of raw dataset respectively, and the query window is from 10 days to 6 months. In particular, we define that the spatial query window is restricted by the MBR of the polygon. For example, spatial window 10 km${}^{2}$× 10 km${}^{2}$ represents a polygon region with latitude range [0,500,1000,900,1000,900,600,400] and longitude range [400,0,100,500,700,1000,900,1000,500]. We evaluate the performance of the STPRQ algorithm from three parameters, i.e., data sizes, spatial window, and time window.

**Figure 6:**
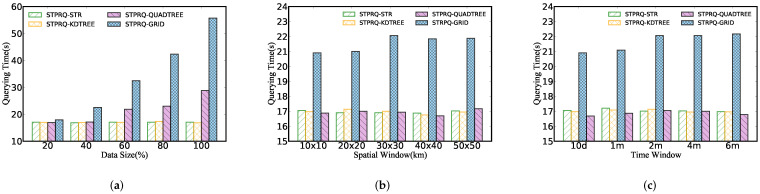
Performance of spatio-temporal polygon range query. (**a**) Data size. (**b**) Spatial window. (**c**) Time window.

Figure 6 performs spatio-temporal polygon range query on different indexes. We can obtain three insights in this figure: (1) The query time increases rapidly with the expansion of data sizes, in large part because the more data that meets the queries are scanned and returned, the more disk IOs are triggered. (2) However, the query performance keeps its stability as spatial and time windows increase. It can be inferred that due to spatial indexes, STPRQ can accurately locate the physical block where the data lies without scanning massive invalid records. (3) Moreover, we can conclude from Figure 6 that the grid index has the worst performance in comparison with other indexes. This is simply because grid index is a one-level flat index that partitions the data according to a grid, which may scan and query more data blocks to some extent.

#### 5.2.2. Comparison of STPRQ and STRQ

**Figure 7:**
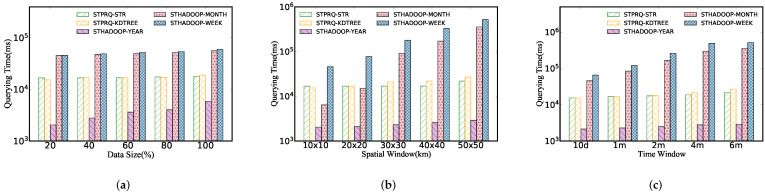
STPRQ Comparison with STRQ in ST-Hadoop. (**a**) Data size. (**b**) Spatial window. (**c**) Time window.

As shown in Figure 7, we describe a performance comparison between the STPRQ algorithm and the spatio-temporal range query (STRQ) algorithm in ST-Hadoop based on different indexes. The phenomenon is as follows.

Different data sizes. Figure 7a manifests the performance of spatio-temporal polygon range query compared with spatio-temporal range query in ST-Hadoop. In comparison with STRQ, STPRQ reduces the response time to query data partitions. This implies that performance in range query depends on the time overhead of scanning and filtering data partition, directly related to response time. This conclusion explains why the year-based ST-Hadoop index takes the least time overhead.

Different spatial windows. From Figure 7b, we observe that the running time in STPRQ maintains the same level as the spatial interval varies when other parameters are fixed, such as data sizes and time windows. This occurs because we simplify building the time index on a spatial index and reduce the partition caused by queries that span a significant time interval. In the majority of cases, our algorithm outperforms ST-Hadoop better in most cases. Nevertheless, note that the performance of the STHadoop−year overwhelms all of the composite indexes. This can be elucidated that the STHadoop-year index takes fewer partitions than STHadoop−month and STHadoop−week. Consequently, the querying delay is relatively small.

Different time windows. Figure 7c demonstrates the results of range query processing under a variety of time intervals. It can be concluded that the increase in the time window has minimal impact on the performance of STPRQ, while STRQ in ST-Hadoop suffers from the varying time. This difference may arise from these reasons: (1) the location of the data partition is first performed, and then relevant records are filtered by time in STPRQ. Since each scan needs to traverse the same time range of data, the time window slightly influences its performance; (2) in contrast, the performance of STHadoop depends on time intervals. When the time gradually becomes more extensive, ST-Hadoop scans more time slices based on data blocks.

#### 5.2.3. Performance of Air Traffic Flow Statistic

Timely and efficient air traffic flow statistics play a significant role in improving the accuracy of air traffic flow management (ATFM), providing assistance to support future, more intelligent flight scheduling strategies. The statistical information can provide assistance to reflect the problem of air collision in the air traffic control (ATC). Air traffic flow statistics aim to calculate the number of aircraft within specific airspace over a certain time period. The traditional approaches of calculating such tasks show their weakness in two parts: (1) they fail to capture the features of complicated three-dimensional time-dependent airspace, and (2) they are not optimized to deal with large-volume spatio-temporal data covering high-dimensional features. Spatio-temporal range queries have advantages in calculating the eligible flow records.

Therefore, utilizing the spatio-temporal polygon range query algorithm, we further propose a traffic flow statistical strategy for civil aviation airspace traffic. We collected large-volume ADS-B data from September 2018 to March 2019 with storage of 70.6 GB in total.

**Figure 8:**
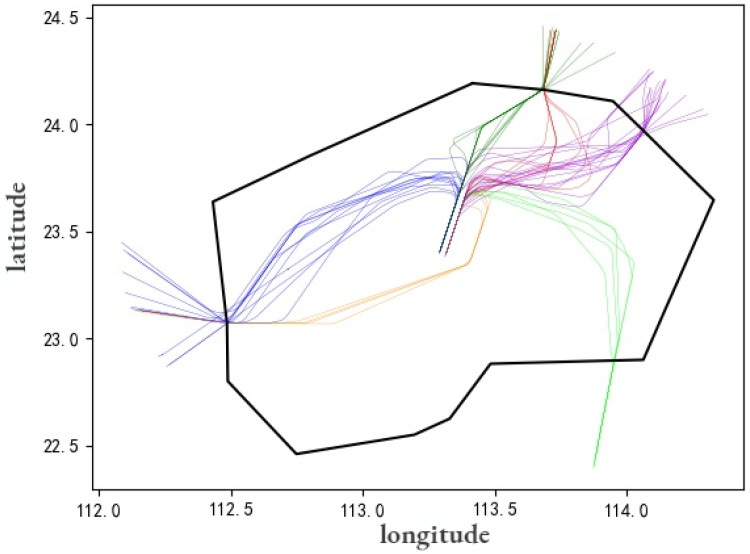
The distribution of ADS-B tracks of inbound flights in the terminal area of Guangzhou Baiyun Airport (ZGGG).

**Figure 9:**
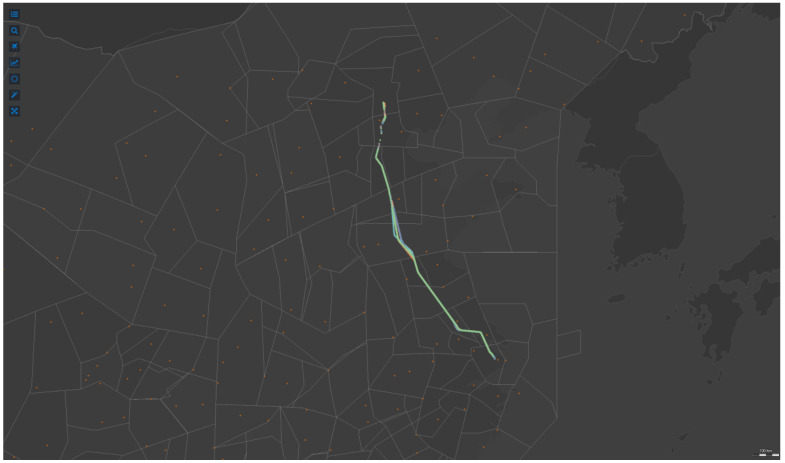
The flight trajectories of the urban air route from Beijing (ZBAA) to Shanghai Hongqiao (ZSSS).

Figure 8 shows the distribution of ADS-B tracks of inbound flights in the terminal area of Guangzhou Baiyun Airport (ZGGG). Diverse colors represent inbound flight track bundles in different directions, and different tracks bundles correspond to different approach sectors. Figure 9 presents the flight trajectories of the urban air route from Beijing (ZBAA) to Shanghai Hongqiao (ZSSS). The flight trajectories pass through regional sectors which are charged by different regional ATC Bureau, and the aircraft flies according to the planned path. The corresponding light gray polygon on the way represents the current airspace sector.

To make a description and analysis without loss of generality, we select several arbitrary sectors from typical busy air routes, i.e., the Nanjing–Beijing route. Considering making a comprehensive analysis and reasonable comparisons, we choose three sectors within the Nanjing–Beijing route. Based on the approximately one million items of ADS-B data in six months, we carried out a traffic flow statistic for several sectors defined by the air traffic control department. At first, we store ADS-B trajectory data and physical airspace sector data on the Hadoop cluster. Then, to utilize an available dataset for our further experiments, we implement some data preprocessing methods on the huge amounts of data obtained. In the end, with the spatio-temporal polygon range query operation built as above, air traffic flow statistic based on ADS-B messages is provided.

**Figure 10:**
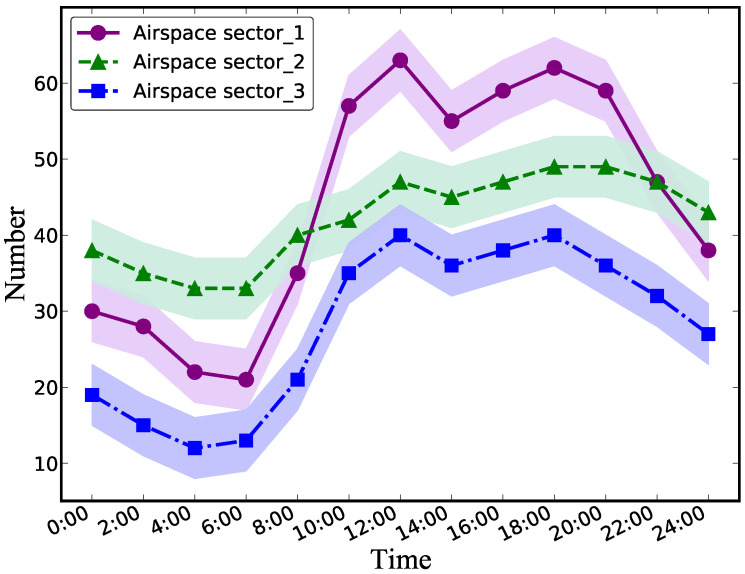
The air traffic flow results of different sectors within the Nanjing–Beijing route.

The statistical hourly number of different sectors inside the Nanjing–Beijing route is depicted in Figure 10. We also characterize the confidence interval to indicate the authenticity and volatility of the results. As illustrated in Figure 10, the air traffic volume of the three airspaces follows a similar periodic law, and the statistical traffic of these routes all present apparent peaks and valleys. In the early morning hours, the route traffic is very sparse. It drops to the bottom at 6:00 and peaks at about 12:00 and 18:00. It is rather remarkable that the area of the green line is relatively moderate. This phenomenon is because this sector is presumably close to the airport terminal area, where traffic is comparatively large and stable.

### 5.3. Experimental Analysis of Spatio-Temporal kNN Query

#### 5.3.1. Performance of Spatio-Temporal kNN Query

**Figure 11:**
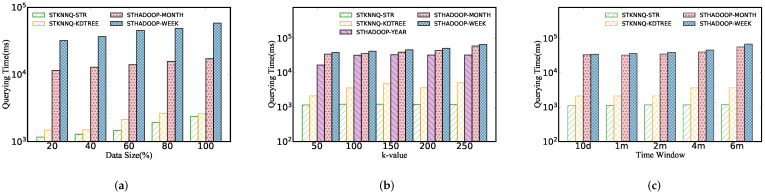
Performance of spatio-temporal kNN query. (**a**) Data size. (**b**) *K*-value. (**c**) Time window.

Different data sizes. As shown in Figure 11a, with the continual growth of data size, it takes more time to answer a kNN query because, in each expansion process of the kNN query, we trigger a spatio-temporal range query, which scans more records.

Different *k* values. Figure 11b compares kNN query processing performance between our proposed algorithm and ST-Hadoop algorithm. It is relatively straightforward to observe the following:

- The varying *k* makes a slight difference to query performance. We learn that our proposed algorithm keeps steady performance regardless of the parameter change from this result.
- Although ST*k*NNQ and ST-Hadoop kNN query are based on the 100% of the dataset, they achieve a magnitude improvement concerning ST-Hadoop because it is expensive for ST-Hadoop to start a MapReduce job.

Overall, we conclude that our ST*k*NNQ overwhelms ST-Hadoop on kNN query processing time due to the efficiency of the range query mentioned before.

Different time windows. Figure 11c depicts that our method takes more time because a larger time window means more qualified records. However, we do not observe significant differences in the response time between 10 days and two months. A possible explanation is that the *y*-axis is in log scale, and time resolution is in milliseconds. Simultaneously, the performance gap between STHadoop-month and STHadoop-week is almost negligible.

#### 5.3.2. Performance of AIRO Algorithm

**Figure 12:**
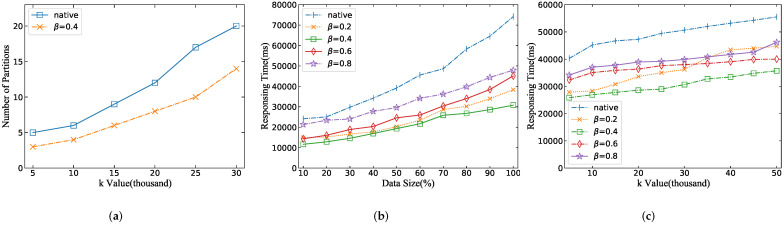
Performance of AIRO algorithm. (**a**) depicts the comparison of the number of partitions for the native algorithm and the optimized algorithm as the value of *k* increases; (**b**) shows the impact of the range radius factor β on response time for different dataset sizes; and (**c**) indicates the influence of β on response time under different *k* values. The different values of β reveal how the radius of the iteration range changes. The larger the value of β, the faster the growth rate of the iteration range radius. (**a**) Data size. (**b**) *K*-value. (**c**) Time window.

In order to test the effectiveness of the AIRO algorithm, we conduct the following experiments to verify the influence of the range radius factor β on the number of query data partitions and the response time of ST*k*NNQ algorithm. It can be observed from Figure 12a that the number of partitions of the optimized algorithm is significantly less than the number of original query partitions, which verifies the effectiveness of the AIRO algorithm. Figure 12b shows that the responding time for all methods increases progressively as the number of data increases. This is because as the data size grows, so does the number of queryable partitions. Correspondingly, data records are more widely distributed, and the iterative range needs to be expanded to be traversed. We can find that the original algorithm has a higher responding time than other algorithms because it scans redundant data blocks, which proves the excellent effect of the AIRO algorithm in reducing response time.

In addition, it is observed that the algorithm’s performance is best when β=0.4. The explanation for this phenomenon is that β=0.4 makes a trade-off between data partitions and iteration times, which means reducing the probability of querying unnecessary data blocks and avoiding the overhead of initializing MapReduce tasks caused by multiple iterations. However, β=0.8 denotes that the query algorithm will query a large number of data blocks at the beginning. It can obtain better performance in the case of small data size, but as the number of data increases, the algorithm will gradually degenerate into the original algorithm to cause poor performance.

From Figure 12c we can conclude the following: (1) With the constant increase of the k value, the response time of the query algorithm tends to be stable. (2) We can also observe that when β=0.4, the response time of the algorithm was the shortest, and the query effect was better than the performance of β=0.2 and β=0.8. Of course, different data distribution and indexing techniques will affect the actual effect of the experiment. (3) It is worth noting that the performance at β=0.4 is not always stable but increases rapidly. When the value of *k* is small, we can achieve a good query effect with a small number of data partitions. However, as the value of k increases, it is very likely that the size of the value of k exceeds the number of records in the existing partition. The smallest granularity of step size will lead to a slow iteration range; not only does it not increase the number of new data blocks but it also brings the overhead of loading the job running resources caused by each iteration, which is intolerable in the query algorithm.

## 6. Conclusions

In order to solve the multi-dimensional spatio-temporal sensing data query problem in complex query scenarios and improve the efficiency of spatio-temporal data query, in this paper, we creatively propose a spatio-temporal polygon range query (STPRQ) algorithm, which aims to find all records from a polygonal location in a given time interval. Then, we present a novel ST*k*NNQ algorithm to directly search the *k* nearest neighbors of a given object. To optimize the ST*k*NNQ algorithm, we further propose an adaptive iteration range optimization (AIRO) algorithm. Finally, extensive experiments based on ADS-B trajectory datasets demonstrate that our query processing algorithms can significantly reduce response time over baseline algorithms. The STPRQ algorithm proposed in this paper proved to be reliable for improving the efficiency of air traffic flow statistics. The limitation of our work is that we consider point data only. There are two main directions to polish this work. First, a novel data partitioning framework would be performed to efficiently perform over large spatio-temporal data with any geometry types. Second, regarding AIRO algorithm, there are still some parameters, i.e., geometry types, or spatio-temporal distributions of datasets, that can have effects on iteration range. It is not easy to consider all parameters for every kNN manually. As a result, we can develop an query optimization cost model to minimize the responding time by reducing the number of partitions that contain the final answer for each of its operations.

## Figures and Tables

**Table 1:** The most representative DSDMSs with functional comparisons.

DSTDMS	Architecture	Query Operation
Hadoop-GIS	Hadoop	Range query, spatial join
SpatialHadoop	Hadoop	Range query, kNN, spatial join
ST-Hadoop	Hadoop	ST-range query, ST-join, kNN
Hadoop-Trajectory	Hadoop	Pass, Traj, WindowIntersect
Simba	Spark	Range query, kNN, spatial join
SpatialSpark	Spark	Range query, spatial join
GeoSpark	Spark	Range query, kNN
STARK	Spark	Range query, kNN, spatial join
JUST	NoSQL	ST-range query, kNN

**Table 2:** An example of spatio-temporal record.

Record	Spatio-Temporal Properties			Other Properties		
$r_{1}$	$lng_{1}$	$lat_{1}$	$time_{1}$	$height_{1}$	$speed_{1}$	$angle_{1}$
$r_{2}$	$lng_{2}$	$lat_{2}$	$time_{2}$	$height_{2}$	$speed_{2}$	$angle_{2}$
…	…	…	…	…	…	…
$r_{i}$	$lng_{i}$	$lat_{i}$	$time_{i}$	$height_{i}$	$speed_{i}$	$angle_{i}$

**Table 3:** The descriptions of datasets.

Attributes	ADS-B Trajectory Data	Synthetic Data
Records	10 million	100 million
Raw size	379 MB	10.3 GB
Timespan	1 January 2019–1 July 2019	1 September 2018–1 September 2019

**Table 4:** The configuration of query parameters.

Parameters	Settings
Data size (%)	20, 40, 60, 80, 100
Time window	10 d, 1 m, 2 m, 4 m, 6 m
Spatial window (km${}^{2}$)	10 × 10, 20 × 20, 30 × 30, 40 × 40, 50 × 50
*k* value	50, 100, 150, 200, 250
Factor of range radius (β)	0.2, 0.4, 0.6, 0.8

## Data Availability

Data sharing is not applicable to this article.
